# Enhanced mechanical properties of alkali-activated dolomite dust emulsified asphalt composites

**DOI:** 10.1038/s41598-024-73552-z

**Published:** 2024-12-30

**Authors:** Qun Liu, Weiqing Lin, Lei Xi

**Affiliations:** 1 School of Architecture and Art Design, Hubei Communications Technical College, Wuhan, China; 2 School of Civil Engineering, Architecture and Environment, Hubei University of Technology, Wuhan, China

**Keywords:** Waste dolomite dust, Alkali-activated, Emulsified asphalt, Mechanical property, Civil engineering, Structural materials

## Abstract

The dolomite dust-emulsified asphalt composite (DAC) with excellent mechanical properties was successfully prepared using alkali activation. The effects of different alkali concentrations and emulsified asphalt contents on the mechanical properties of the materials were studied. And the micro-mechanisms of its mechanical performance changes were analyzed through SEM and XRD characterization. The experimental results show that the specimens have excellent mechanical properties: the 7-day compressive strength can reach 76.67 MPa, and the bending and compressive strength ratio is about twice that of silicate-based geopolymer-emulsified asphalt composite (SAC). With an increase in emulsified asphalt content, the compressive strength of the samples decreases, while the bending strength increases first and then decreases. When the emulsified asphalt content is 1%, the bending strength of the sample is up to 28.81 MPa, which is 25% higher than that of the sample without emulsified asphalt. The in-situ formation of calcium carbonate crystal clusters within the DAC was suggested that may support this performance. This crystalline structuring contributes to an expanded interfacial contact area between the asphalt and skeleton particles, thereby enhancing the demulsification and bonding properties of the emulsified asphalt. This indicates that an appropriate emulsified asphalt content can play a toughening role in the system, providing a new idea for designing high-toughness alkali-activated materials.

## Introduction

Ecological and environmental protection is indeed an important aspect of healthy societal development^1^. In China’s industrial field, 85% of the raw materials come from mineral products in mining production. The demand for mineral resources is growing, and the huge mining industry has brought a lot of tailings and waste dust storage^2^. Dolomite is a natural carbonate mineral with abundant reserves, accounting for about 1.7% of the total crust. It is widely used in the field of building materials and metallurgy, but high purity requirements for raw materials are necessary. For example, the MgO content of dolomite used in the metallurgical industry needs to be greater than 20%, and the SiO_2_ content cannot be greater than 3%^3^. Dolomite ore that does not meet the demand is difficult to use in the building materials and metallurgy industry, and the perennial accumulation has become a kind of solid waste that needs to be solved urgently. Therefore, using marginal dolomite as raw materials not only avoids raw material competition with these industries but also can realize the effective use of waste natural resources.

Alkali activation is considered an effective means of treating solid waste and producing green cementing materials. The product formed is called alkali-activated materials(AAM) or geopolymer, which not only has the same or even better mechanical properties as Portland cement, but also the production conditions are more mild^4,5^. Compared with the production process of Portland cement (two grinding and one burning), AAM greatly reduce carbon dioxide emissions and energy consumption. At present, the precursor of AAM is usually amorphous aluminosilicate calcined at high temperature, such as metakaolin, fly ash and slag^6–9^. In particular, the research of bulk solid waste mainly fly ash and slag as the precursor of AAM has become a research hotspot at home and abroad, showing great development potential. Lu et al.^10^ used sodium tert-butanol, an organic strong alkali as an activator for preparing fly ash geopolymer to improve their mechanical properties. Bai et al.^11^ prepared high-strength geopolymers with red mud and Class 1, 2, and C fly ash rich in active substances in the presence of an alkali activator. However, the researches on the cheap natural carbonate minerals with huge reserves and the wastes in the mining process as the precursors of AAM is still limited. In current relevant studies, dolomite (CaMg(CO_3_)_2_) is generally considered to lack the gellability because it does not contain the tetracomorate Al of active aluminosilicate precursors, and the research in the field of AAM is very limited^12^. Although some scholars use dolomite as a raw material for the preparation of AAM, dolomite is often used only as an additive or replacement material for traditional aluminosilicate cementing materials. Yip et al.^13^ added 17–35 mm fine limestone and dolomite powder to metakaolin-based geopolymer, respectively, and found that when the addition of limestone or dolomite powder was 20% of the total weight, the compressive strength of geopolymer increased, although it would shrink within 90 days of curing time. But, when the replacement weight is more than 20% of the total weight, the compressive strength decreases due to the interference of the powder on the geopolymer gel network. Szybilski et al.^14^ found that the amount of cement hydration products increased with the increase of dolomite content in cement-based materials. This is due to the fine dolomite particles after crystallization and better water absorption. Mikhailova et al.^15^ showed that the mechanical properties of the composite cementing material could be improved when fine dolomite was added to cement with 20%~25% mass fraction. There are fewer studies on using dolomite as the precursor of alkali excitation materials. Aizat et al.^16^ studied the influence of different NaOH molar concentrations on the compressive strength of the dolomite base polymer and found that geopolymer obtained when NaOH is 20 M has the highest strength, but it is only 5 MPa, which is difficult to meet the application requirements in construction engineering. Yin Suhong^17^ has deeply explored the properties and mechanism of alkali-activated carbonate ore cement grouting materials, but the strength of the materials is only 1–4 MPa, which is mainly used for the reinforcement and seepage prevention of sandy soft strata and has not been applied in civil engineering structural materials.

Asphalt is a major concrete binding material and has become the preferred material for modern expressways due to its improved driving comfort, safety, and service life of highway pavement^18^. Currently, asphalt is used in the form of hot asphalt, diluted asphalt, and emulsified asphalt. Compared to hot asphalt and diluted asphalt, emulsified asphalt can save 40–50% energy, improve construction conditions, reduce project costs by more than 20–30%, and effectively reduce excessive aging of asphalt caused by high temperature heating. It also reduces the amount of carcinogenic benzopyrazine that is released through volatilization^19^. But the general emulsified asphalt composite has the technical problem of insufficient strength in the early stage^20^. Combining it with AAM can not only improve the shortcomings of low toughness and high brittleness of AAM, but also effectively solve the problem of low early compressive strength of general emulsified asphalt composites. In addition, The combination of alkali-activated dolomite and emulsified asphalt is a novelty exploration, which provides a new way to solve the utilization of waste dolomite dust.

In this study, the abandoned dolomite dust from a quarry was used as the precursor of the consolidated phase (i.e., alkali-activated material). Emulsified asphalt was used as the viscoelastic phase, while sodium hydroxide and sodium silicate were used as alkali-activated agents. The abandoned dolomite dust-emulsified asphalt composite material with excellent mechanical properties was prepared by compression molding. The effects of different alkali concentration and emulsified asphalt contents on the compressive strength and bending strength of the sample were investigated, and the different properties of the emulsified asphalt-silicate based AAM and the emulsified asphalt-dolomite based AAM were compared in order to provide a new idea for the preparation of AAM with high bending resistance.

## Experimental

### Materials

**Fig. 1:**
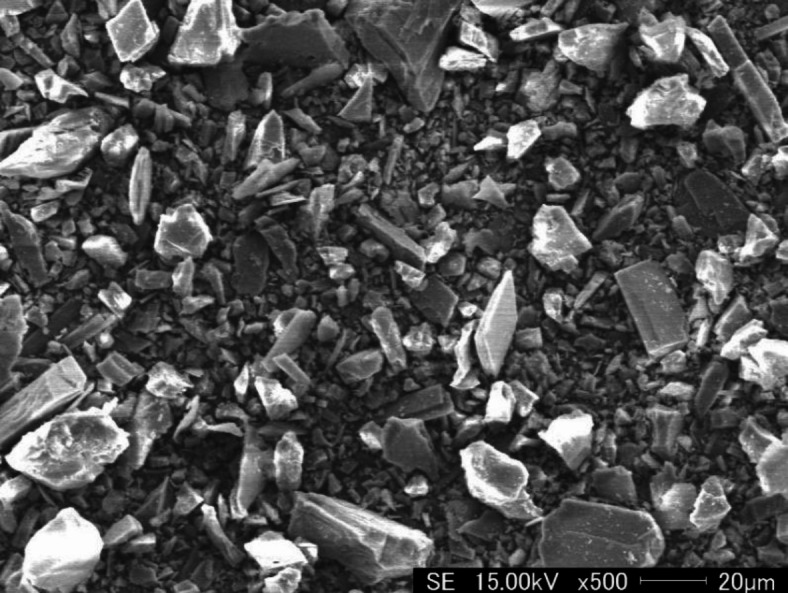
The microscopic morphology of waste dolomite dust.

**Fig. 2:**
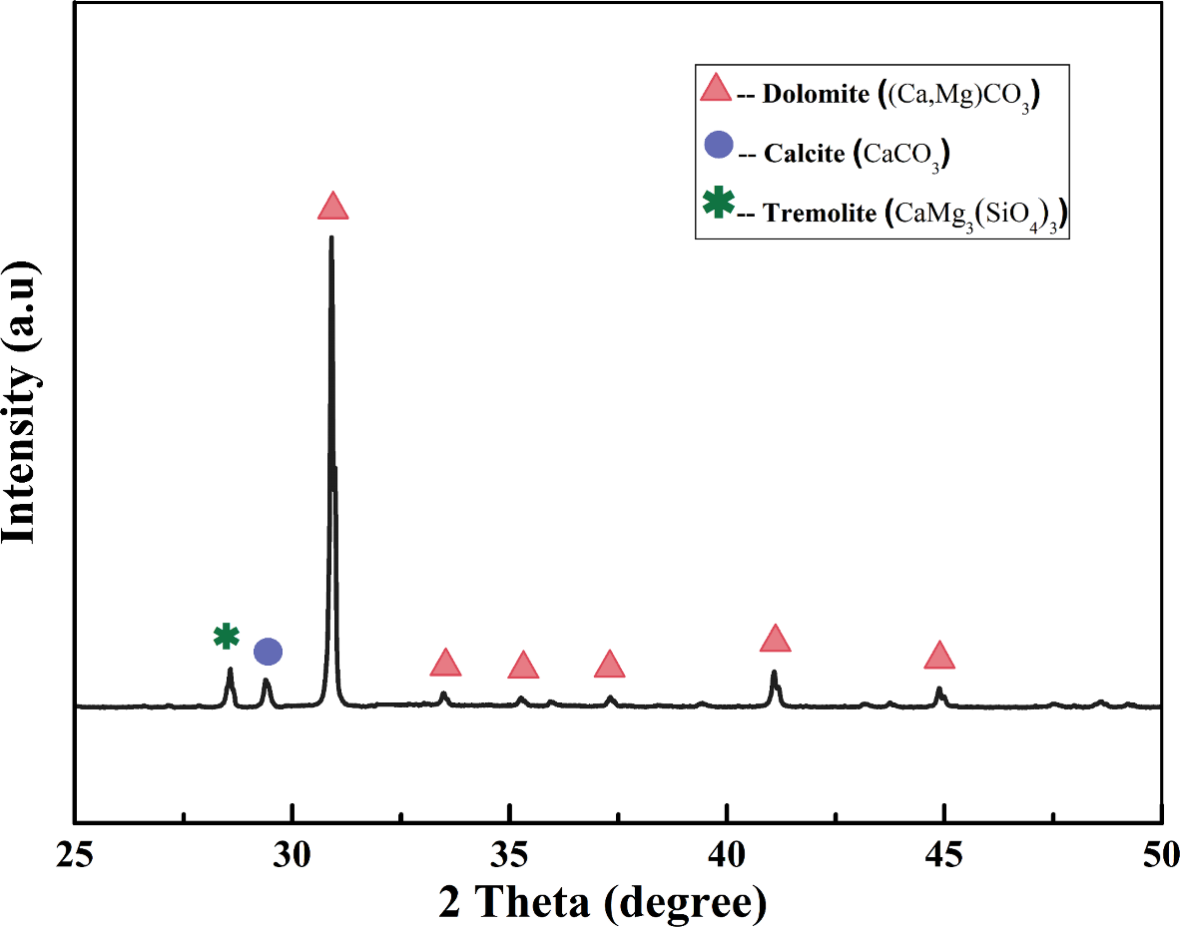
XRD pattern of waste dolomite dust.

70#A grade road asphalt was selected from Sinopec Maoming Branch and JY-C2 cationic medium crack emulsifier from Jiangsu Jinyang New Material Technology Co., LTD. Dolomite powder with a particle size of 250 mesh was obtained from the abandoned dolomite powder produced during the mining operation in a quarry in Shiyan, Hubei, China. Its microscopic morphology was examined using the field emission scanning electron microscope (Nova NanoSEM 450, FEI, Netherlands), as shown in Fig. 1. Its chemical composition was determined by X-ray fluorescence (XRF-1800, Shimadzu Company, Japan), and the results were shown in Table 1. Mineralogical analysis was carried out by X-ray diffractometer (Empyrean, Panaco, Netherlands), as shown in Fig. 2. The main phases were dolomite, with some doped calcite and tremolite phases. The alkali activator consists of sodium hydroxide particles and liquid sodium silicate. The modulus of sodium silicate is 3.1, the content of silica (SiO_2_) is 27.66%, and the content of sodium oxide (Na_2_O) is 8.8%. Sodium hydroxide particles are analytically pure (AR) grade.

**Table 1:** Chemical composition of waste dolomite dust (wt%).

CO_2_	CaO	MgO	SiO_2_	Al_2_O_3_	Fe_2_O_3_	Na_2_O	K_2_O	P_2_O_5_
45.22	24.31	24.11	5.58	0.53	0.19	0.03	0.02	0.01

Table 2 shows the raw materials used in emulsified asphalt. First, the asphalt was heated in an oven at 165℃ until it was fully flowing. Meanwhile, a mixture containing emulsifier at 0.9% of the total mass and water at 51.1% of the total mass was heated and stirred on an induction oven until the emulsifier was completely dissolved in the water. Then, the colloidal mill was preheated and the mixture of emulsifier and water was added. After that, the colloidal mill was started for 15 s and the asphalt at 48% of the total mass was slowly poured into the outlet of the funnel pipeline while constantly stirring it. Once all the asphalt is added, the colloid mill was started again for 40 s. Finally, the emulsified asphalt was filtered by a screen with an aperture of 1.18 mm and was stored for reserve.

**Table 2:** Raw material proportioning for emulsified asphalt (wt%).

Name	Asphalt	Water	Emulsifier
JY-C2	48	51.10	0.90

### Sample preparation

First, add X g of dolomite powder to a grinding bowl. Then, add 1%X g, 1.5%X g, 2%X g, and 3%X g of sodium hydroxide particles and 10%X g of water glass to each experimental group and mix them evenly with the powder. When the powder is wet, add 1%X g, 3%X g, and 5%X g of emulsified asphalt that has been prepared to each experimental group and mix them evenly. Fill the mixed powder into a special steel mold and press it under a load of 75 MPa for 1 min to form it. The mold was removed to obtain the compressive strength sample with diameter of 10 mm and height of 10 mm and the bending strength sample with diameter of 10 mm and height of 25 mm. Three parallel samples with the same preparation parameters were selected for each group of tests. The average value of the three parallel specimens was taken as the test result of each group.

The annular test mold (inner diameter 60 mm, thickness 6 mm) was pre-wet with the lino felt base, the emulsified asphalt was poured in and scraped flat, and the specimen was slightly formed after being placed for 1 min for demoulding. The specimen was maintained at 25℃ and 95% humidity for 60 min, and two cohesion specimens were prepared for each mix ratio.

### Test and characterization

The samples underwent compressive strength and bending strength tests using a universal testing machine (Matest Industrial Systems (China) Co., Ltd.). The loading method was displacement loading at a rate of 2 mm/min. The field emission scanning electron microscope (Nova NanoSEM 450, FEI, Netherlands) was used to obtain the microstructure of the sample at 0.5 nA using a scattered electron detector.

After the preparation of emulsified asphalt, the pH value was determined with a digital pH meter, and the samples were respectively tested by particle ion charge test, storage stability test and Angler viscosity test according to the asphalt related test procedures JTG E20-2011 T 0754-2011, to complete the performance analysis and determination of emulsified asphalt samples.

The cohesion test was carried out in accordance with the requirements of the specification JTG E20-2011 T0754-2011. The cohesion test specimen was prepared after completing the repeatability test of the cohesion test instrument, and was placed for 1 min to make it slightly formed and then released for maintenance. The test was carried out after the specified time.

## Results and discussion

Table 3 shows the basic property parameters of the emulsified asphalt. The results indicate that the emulsified asphalt is cationic acidic emulsified asphalt with an Angler viscosity of 10.95 at 25℃ and a storage stability of 0% at (15 ± 1) ℃ for 24 h. These findings suggest that the emulsified asphalt has high viscosity and stable storage stability.

**Table 3:** Basic properties of emulsified asphalt.

Types	Ion species	pH	Angler viscosity	Storage stability (%)	Cohesion (Nm)
Medium breaking	cation	2.98	10.95	0	1.28

**Fig. 3:**
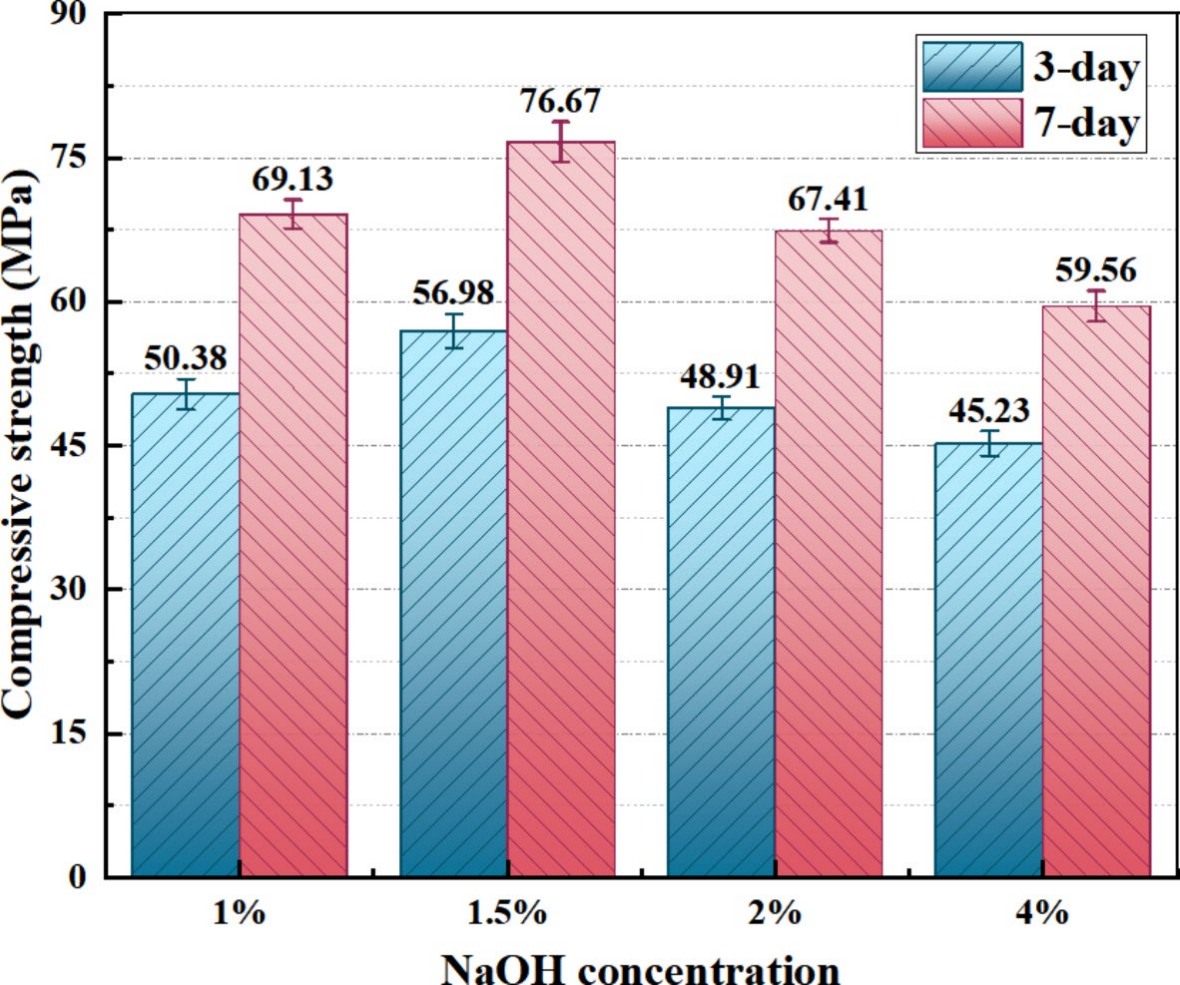
Effect of different alkali concentration on the compressive strength.

Figure 3 illustrates the variation in compressive strength of the alkali-activated dolomite samples without emulsified asphalt addition as a function of the NaOH content. The purpose of this is to determine the optimal NaOH content, which will serve as the basis for subsequent studies investigating the effects of different emulsified asphalt contents on the sample properties. As shown in Fig. 3, the compressive strength of the sample after 7 days increased initially and then decreased with the increase of alkali concentration. The compressive strength of the sample was highest at 76.67 MPa when the concentration was 1.5%, indicating that there is an optimal alkali concentration that makes the sample have more excellent mechanical properties. At the same time, when the alkali concentration was between 1% and 2%, the compressive strength of the sample after 7 days was not less than 65 MPa. However, more and more attention has been paid to the toughness of building materials at this stage. To further improve the flexural performance of AAM is conducive to broadening its use range and increasing its safety factor. Therefore, the effect of emulsified asphalt and whisker toughening will be further discussed in the following sections.

**Fig. 4:**
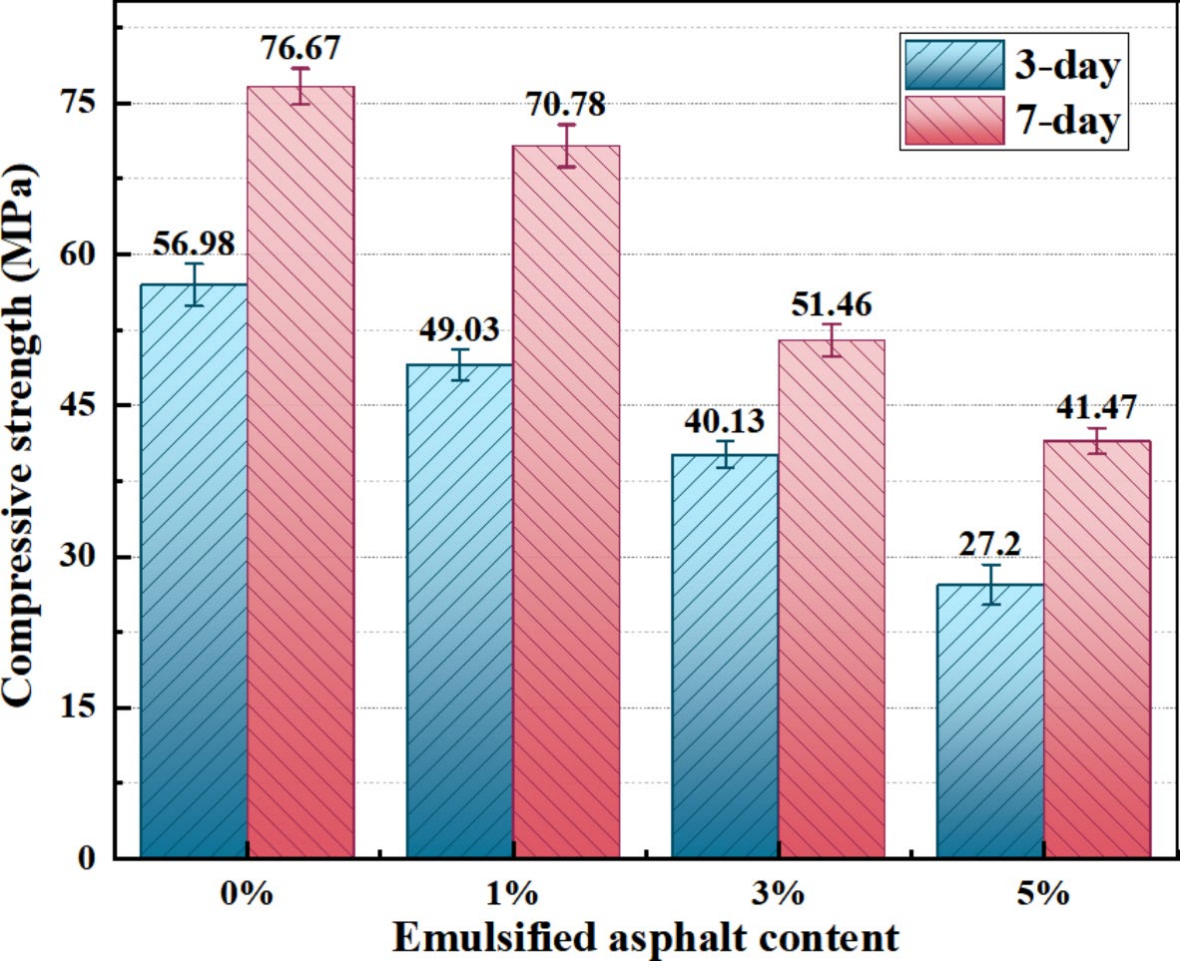
Effect of emulsified asphalt content on compressive strength.

Figure 4 shows the relationship between the amount of emulsified asphalt added and the compressive strength of samples after 3 and 7 days. The NaOH concentration of all samples is 1.5%. As the amount of emulsified asphalt added increases, the compressive strength of samples after 3 and 7 days shows a downward trend. This is because asphalt, as a toughening phase, is filled in the skeleton system of alkali-activated dolomite consolidated phase, and its contribution to the compressive strength of samples is weaker than that of gel produced in alkali-activated dolomite reaction. Therefore, as the amount of emulsified asphalt increases, the compressive strength of samples shows a downward trend. Comparing the strength of different asphalt contents after 3 and 7 days, it can be found that with the increase of emulsified asphalt content, the growth rate of sample strength with age increases. This indicates that the addition of emulsified asphalt slows down the reaction rate of alkali-activated dolomite and its strength development rate.

**Fig. 5:**
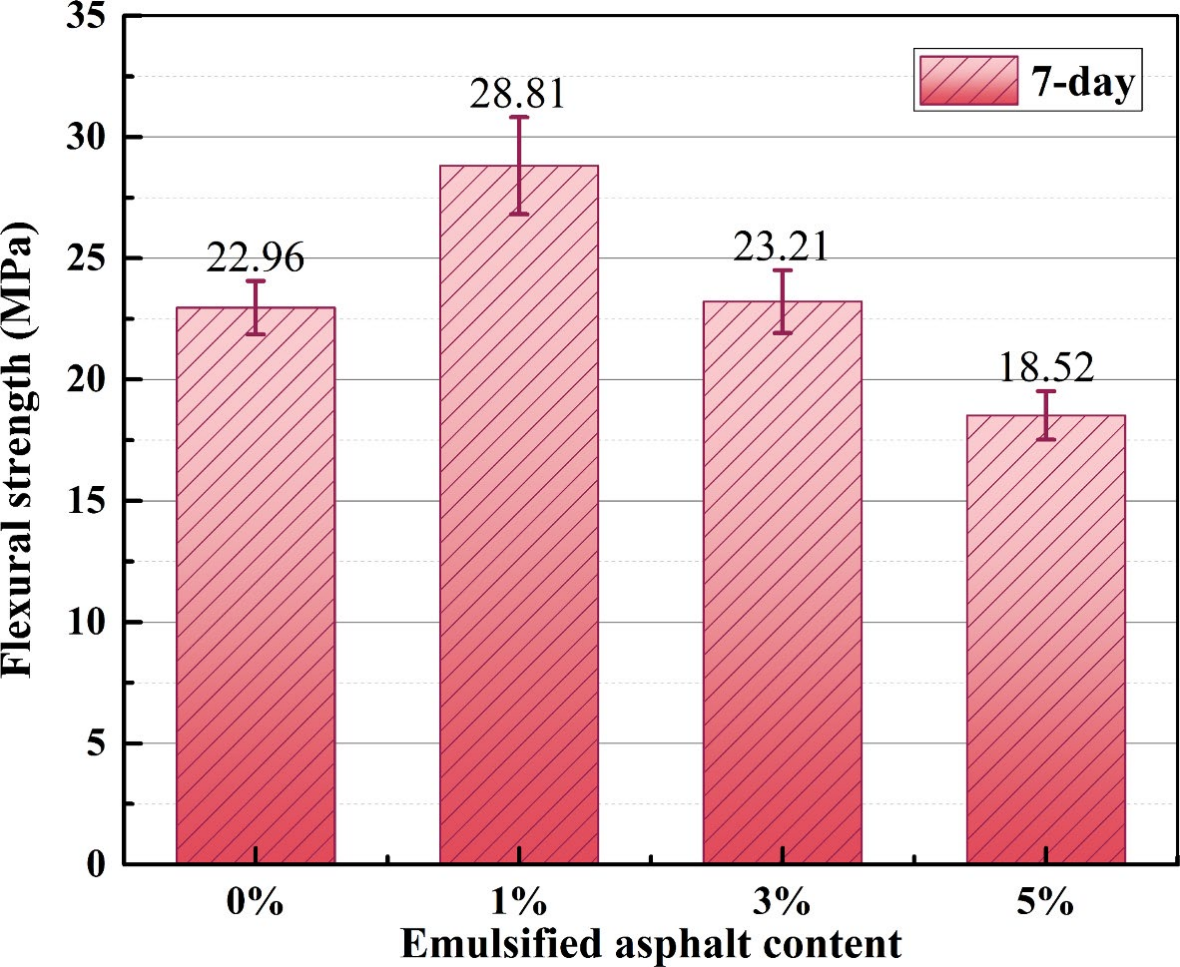
Effect of emulsified asphalt content on the bending strength.

Figure 5 shows the influence of different emulsified asphalt contents on the bending strength of the sample. The NaOH concentration of all samples is 1.5%. With an increase in emulsified asphalt content, the 7-day compressive strength of the sample first increased and then decreased. When the emulsified asphalt content was 1%, the bending strength of the sample was the highest, which was 25% higher than that of the sample without emulsified asphalt. It can be seen from the error bar analysis that the data discreteness of the test results is small and the data stability is high. This indicates that emulsified asphalt with an appropriate dosage plays a toughening role in alkali-activated dolomite consolidation systems and improves their fracture resistance. This provides a new idea for designing AAM with higher toughness in the future.

The bending and compressive strength ratio (B/C) is an important indicator of materials’ intrinsic mechanical properties. Selecting an appropriate strength ratio is crucial for ensuring structural safety and achieving high-quality processing. According to relevant literature^21,22^, the B/C of cement-based materials is typically about 10%~15%, while the B/C of SAC is about 15%~20%. These materials exhibit significant brittleness characteristics. Additionally, when considering data from Figs. 4 and 5, it can be observed that the B/C of DAC is approximately 40%~45%, indicating that DAC has better fracture properties than SAC and cement-based materials. This is mainly due to differences in reaction mechanism and microstructure of reaction products between the silicate-based material and dolomite in alkali activator solution.

**Fig. 6:**
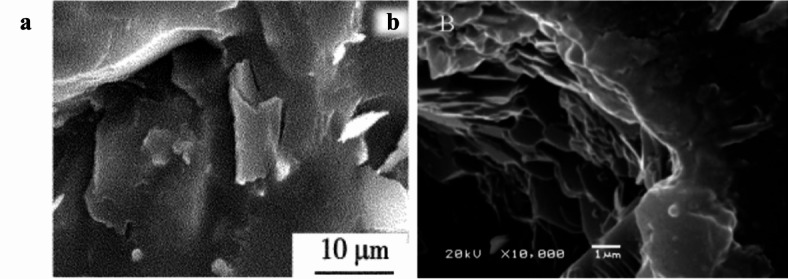
SEM diagram of silicate-based geopolymers: (**a**) Slag-based geopolymer^25^; (**b**) Metakaolin-based geopolymer^26^.

Silicate-based geopolymers dissolve and condense in alkaline environments, as shown in Eqs. (1) and (2)^23,24^. The sample section’s micro-morphology is uniform and continuous, as seen in Fig. 6a and b below.

1 $$\left( {{\text{Si}}_{2} {\text{O}}_{5} ,{\text{Al}}_{2} {\text{O}}_{2} } \right)_{n} + n{\text{H}}_{2} {\text{O}}\xrightarrow{{{\text{NaOH}}}}n\left( {{\text{OH}}} \right)_{3} - {\text{Si}} - {\text{O}} - {\text{Al}}^{ - } \;\left( {{\text{OH}}} \right)_{3}$$

2 $$n\left( {{\text{OH}}} \right)_{3} - {\text{Si}} - {\text{O}} - {\text{Al}}^{ - } \;({\text{OH}})_{3} \xrightarrow{{{\text{NaOH}}}}{\text{Na}}^{ + } \;\left( {{\text{SiO}}_{2} ,\;{\text{AlO}}_{2} } \right)_{n} + 3n{\text{H}}_{2} {\text{O}}$$

**Fig. 7:**
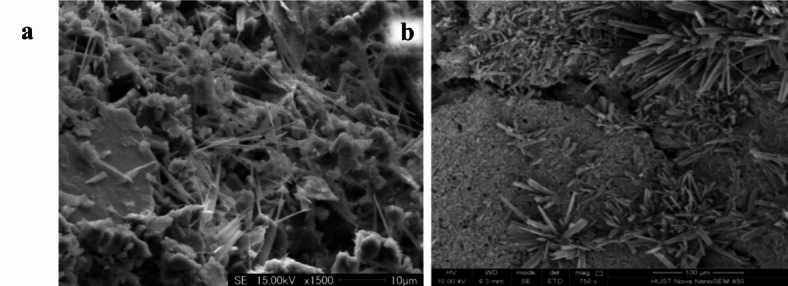
SEM diagram of dolomite-based geopolymers: (**a**) compression molding^16^, (**b**) casting molding.

**Fig. 8:**
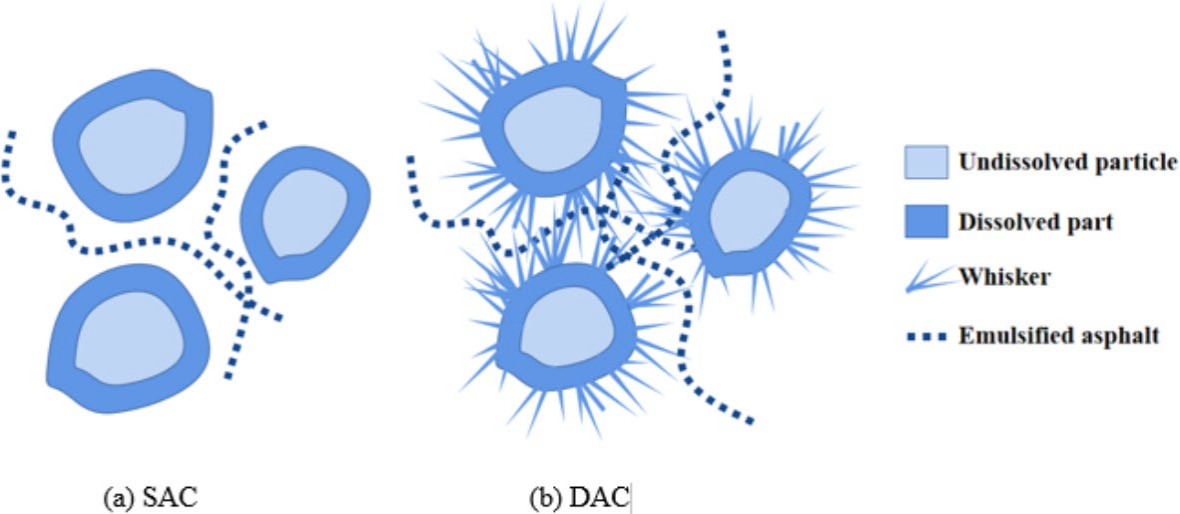
Microstructure diagram of two different emulsified asphalt-AAM composites.

According to previous studies^27,28^, dolomite dust mainly undergoes dedolomitization reaction (Eq. 3) and ion crosslinking reaction (Eq. 4) to produce silicate gel in an alkali environment, making the sample produce strength. This includes a reaction process of dissolution and precipitation, during which the dolomite forms calcium carbonate in clusters of crystals, as shown in Fig. 7a,b below. Figure 7a,b are respectively the micrographs of alkali-activated dolomite samples prepared by compression molding and casting molding without emulsified asphalt. Unlike the homogeneous and continuous product obtained in Fig. 6, the calcium carbonate obtained in the form of clusters of crystals enables more micropores to be formed in the alkali-activated dolomite sample than in the silicate-based geopolymer. The micropores of these reaction products provide a larger specific surface area for the demulsification of emulsified asphalt so that the asphalt can be more closely coated on the surface of inorganic particles, forming a ductile interpenetrating bond inside the skeleton. The microstructure diagram of two different emulsified asphalt-AAM composite materials is shown in Fig. 8. It can be seen that the DAC samples have more complex morphology of micro-reaction products, and the emulsified asphalt is interwoven in the acicular whisker. The whisker and emulsified asphalt act as a toughening agent together. Therefore, under the same compressive strength, DAC shows better bending performance.

3 $${\text{CaMg}}\left( {{\text{CO}}_{3} } \right)_{2} + 2{\text{NaOH}} = {\text{CaCO}}_{{\text{3}}} \downarrow + {\text{Mg}}\left( {{\text{OH}}} \right)_{2} \downarrow + {\text{Na}}_{2} {\text{CO}}_{3}$$

4 $${\text{(Ca,}}\;{\text{Mg)CO}}_{3} + 2{\text{NaOH}} + {\text{H}}_{{\text{4}}} {\text{SiO}}_{{\text{4}}} = ({\text{Ca,}}\;{\text{Mg)H}}_{{\text{2}}} {\text{SiO}}_{{\text{4}}} + {\text{Na}}_{2} {\text{CO}}_{3} + 2{\text{H}}_{2} {\text{O}}$$

**Fig. 9:**
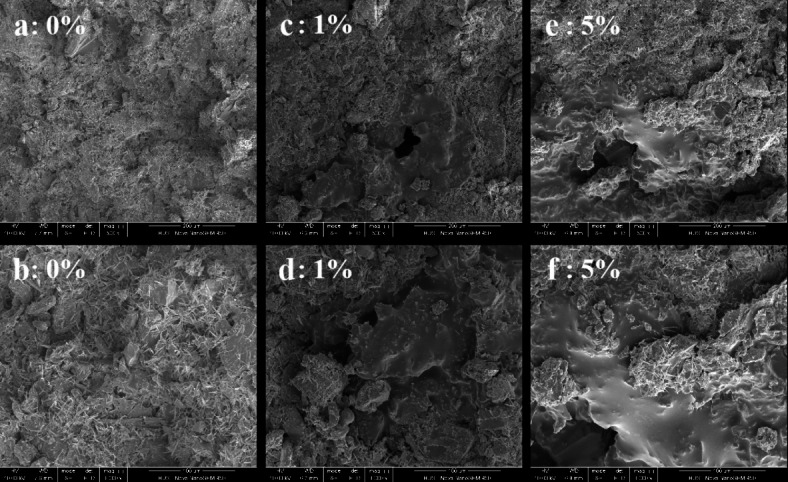
SEM diagram of samples with different emulsified asphalt content.

Figure 9 displays the micromorphologies of samples with different emulsified asphalt content at 500 and 1000 ratios. Samples without emulsified asphalt (a and b) have dense internal structures that include undissolved particles, flocculent gel on particle surfaces, and widely distributed acicular whiskers. The acicular whiskers grown on the surface of the particles are interwoven in the interstitial space between the particles and play a role in connection enhancement. It can not only improve the compressive strength of the sample, but also effectively improve the fracture toughness of the sample. In addition to particles, gels, and whisker components, samples (c and d) containing 1% emulsified asphalt also sporadically distribute demulsified asphalt internally. The demulsified asphalt covers some dolomite particles and plays a certain ductile connection role, resulting in better bending strength for the corresponding samples. However, with an increase in emulsified asphalt content to 5% (c and f), a large area of asphalt coating appears inside the sample, partially replacing the composition of particles, gels, and whiskers inside the sample. Loose pores caused by emulsified asphalt demulsification and water loss also appear around the asphalt-coated particles. This results in a decrease in macroscopic compressive strength and bending strength for the sample. The above results show that appropriate amount of emulsified asphalt and whisker can toughen together, which is conducive to the improvement of alkali-activated dolomite bending property.

## Conclusion

In this paper, the DAC was successfully synthesized, using alkali-activated waste dolomite dust as the consolidation phase and emulsified asphalt as the toughening phase. The main conclusion is as follows:

1. The compressive strength of DAC is up to 76.67 MPa when the alkali concentration is 1.5%. With an increase in emulsified asphalt content, the compressive strength of the samples shows a downward trend, indicating the addition of emulsified asphalt will partially reduce the compressive strength of the DAC.
2. As the dosage of emulsified asphalt increases, the flexural strength of DAC initially rises and subsequently declines. Notably, when the emulsified asphalt dosage remains below 3%, the flexural strength consistently surpasses that of specimens without emulsified asphalt. At an optimal dosage of 1%, the AAD specimen achieves its maximum flexural strength, reaching 28.81 MPa.
3. The bending and compressive strength ratio of DAC is about 40-45%, which is about twice higher than that of SAC. This improvement is likely due to the formation of calcium carbonate crystal clusters within the DAC, resulting in increased microporosity. Consequently, there is enhanced contact between the asphalt and the surface of the skeleton particles, along with improved demulsification and bonding of emulsified asphalt.

These findings highlight the potential toughening effect of appropriately dosed emulsified asphalt in alkali-activated dolomite consolidation systems, offering a novel approach for designing AAMs with high bending performance.

## Data Availability

All data generated or analysed during this study are included in this published article.
